# Extinction of contextual fear memory is facilitated in TRPM2 knockout mice

**DOI:** 10.1186/s13041-025-01181-2

**Published:** 2025-02-27

**Authors:** Seung Yeon Ko, Do Gyeong Kim, Huiju Lee, Sung Jun Jung, Hyeon Son

**Affiliations:** 1 Hanyang Biomedical Research Institute, Hanyang University, Seongdong-gu, Seoul, 04763 Korea; 2 Graduate School of Biomedical Science and Engineering, Hanyang University, Seongdong-gu, Seoul, 04763 Korea; 3 Department of Physiology, College of Medicine, Hanyang University, Seongdong-gu, Seoul, 04763 Korea; 4 Department of Biochemistry and Molecular Biology, College of Medicine, Hanyang University, Seongdong-gu, Seoul, 04763 Korea; 5 College of Medicine, Hanyang University, 222 Wangsimni-ro, Seongdong-gu, Seoul, 04763 Republic of Korea

**Keywords:** Transient receptor potential melastatin type 2 (TRPM2), Extinction, Fear memory

## Abstract

Transient receptor potential melastatin type 2 (TRPM2) is a nonselective cation channel involved in synaptic plasticity. We investigated its role in contextual fear conditioning and extinction of conditioned fear using *Trpm2-*deficient (*Trpm2*^*−/−*^) mice. *Trpm2*^*−/−*^ mice exhibited reduced acquisition of contextual fear memory during conditioning but had an intact freezing response to conditioning context 24 h after conditioning. They also showed a reduced freezing response to extinction training, indicating facilitated extinction. Consistent with this, infusion of flufenamic acid (FFA), a TRPM2 antagonist, into the dentate gyrus (DG) of the hippocampus in fear-conditioned mice facilitated extinction of contextual fear. The enhanced extinction in *Trpm2*^*−/−*^ and FFA-treated mice was associated with down-regulation of immediate-early genes (IEGs) including *Npas4, c-Fos, Arc* and *Egr1* in the hippocampus after extinction training. Our results indicate that TRPM2 plays a positive role in retention of contextual fear memory by modulating neuronal activity in the hippocampus, and suggest that TRPM2 activity could potentially be targeted to strengthen extinction-based exposure therapies for post-traumatic stress disorder (PTSD).

**Supplementary Information:**

The online version contains supplementary material available at 10.1186/s13041-025-01181-2.

## Introduction

Transient receptor potential melastatin type 2 (TRPM2) is a nonselective cation channel permeable to calcium, sodium, and potassium ions and activated by oxidative stress, ADP ribose, and intracellular calcium (Ca_i_^2+^) [1]. It is abundantly expressed in the central nervous system (CNS), including the hippocampus, cortex, and dorsal root ganglion sensory neurons of the spinal cord [2, 3, 4]. In the CNS, TRPM2 is implicated in calcium dysregulation associated with various neuropsychiatric and neurodegenerative disorders [5].

In the hippocampus, TRPM2 contributes to hippocampal synaptic plasticity [6, 7]. Long-term depression (LTD) is impaired in *Trpm2* deficient (*Trpm2*^*−/−*^) mice, which also exhibit memory impairment, enhanced neuronal intrinsic excitability, and imbalanced synaptic transmission [8]. Fear and fear extinction, which are well-known forms of learning in the hippocampus, are thought to be encoded by distinct sets of kinase signaling pathways and their downstream targets [9]. Neurons that are active during contextual fear conditioning (CFC) are either reactivated or suppressed during extinction [10], and this is directly related to gene expression. Indeed many studies show that the expression of immediate early genes (IEGs) such as *Npas4, Arc, c-Fos* and *Egr1* is associated with both context-dependent fear acquisition and context-dependent fear extinction [11].

Despite ample evidence for the roles of TRPM2 in the brain, and high expression of TRPM2 in the hippocampal dentate gyrus (DG) [12], it is not known whether TRPM2 in this brain region contributes to contextual control of fear expression and/or extinction. In the fear conditioning paradigm, mice learn to express a fear response (i.e., freezing) when exposed to a conditioned context (CS, the chamber environment), which has been previously paired with a noxious unconditioned stimulus (US, electrical foot shock). If the mice are then exposed only to the CS without the US, the previously-acquired fear response will gradually decline, a process known as extinction. This paradigm is relevant to post-traumatic stress disorder (PTSD) and is thought to mimic extinction-based exposure therapies [13, 14].

In this study, we investigated the role of TRPM2 in fear memory extinction using *Trpm2*^*−/−*^ mice and pharmacological blockade. Our results show that *Trpm2* deficiency accelerates the extinction of fear memories, and that this is accompanied by suppression of the expression of IEGs including *Npas4, Arc, c-Fos* and *Egr1*.

## Methods

### Mice

*Trpm2*^*−/−*^ mice were generated and characterized previously [2]. In brief. *Trpm2* heterozygotes (*Trpm2*^*+/−*^) were backcrossed into a C57BL/6J inbred background over 10 generations. Heterozygous breeders were then crossed with each other to generate wild-type (WT, *Trpm2*^*+/+*^), heterozygous (*Trpm2*^*+/−*^) and knockout (*Trpm2*^*−/−*^) littermates, as determined by PCR analysis. All experiments were performed with 8- to 12-wk-old male TRPM2 knockout mice, and age-matched male wild-type littermates served as controls. All animals were maintained under a 12-h light/dark cycle with food and water ad libitum, and all animal experiments followed protocols approved by the Institutional Animal Care and Use Committee of Hanyang University.

### Behavioral assessments

Mice were moved to the testing room 2 h before the start of each behavioral test and acclimated to the room conditions. All tests were conducted during the dark cycle of animal housing, and in random order. After each individual test session, the apparatus was cleaned with 70% alcohol to remove any odor and trace of the previously-tested mouse.

### Contextual fear conditioning and fear extinction

**Fig. 1:**
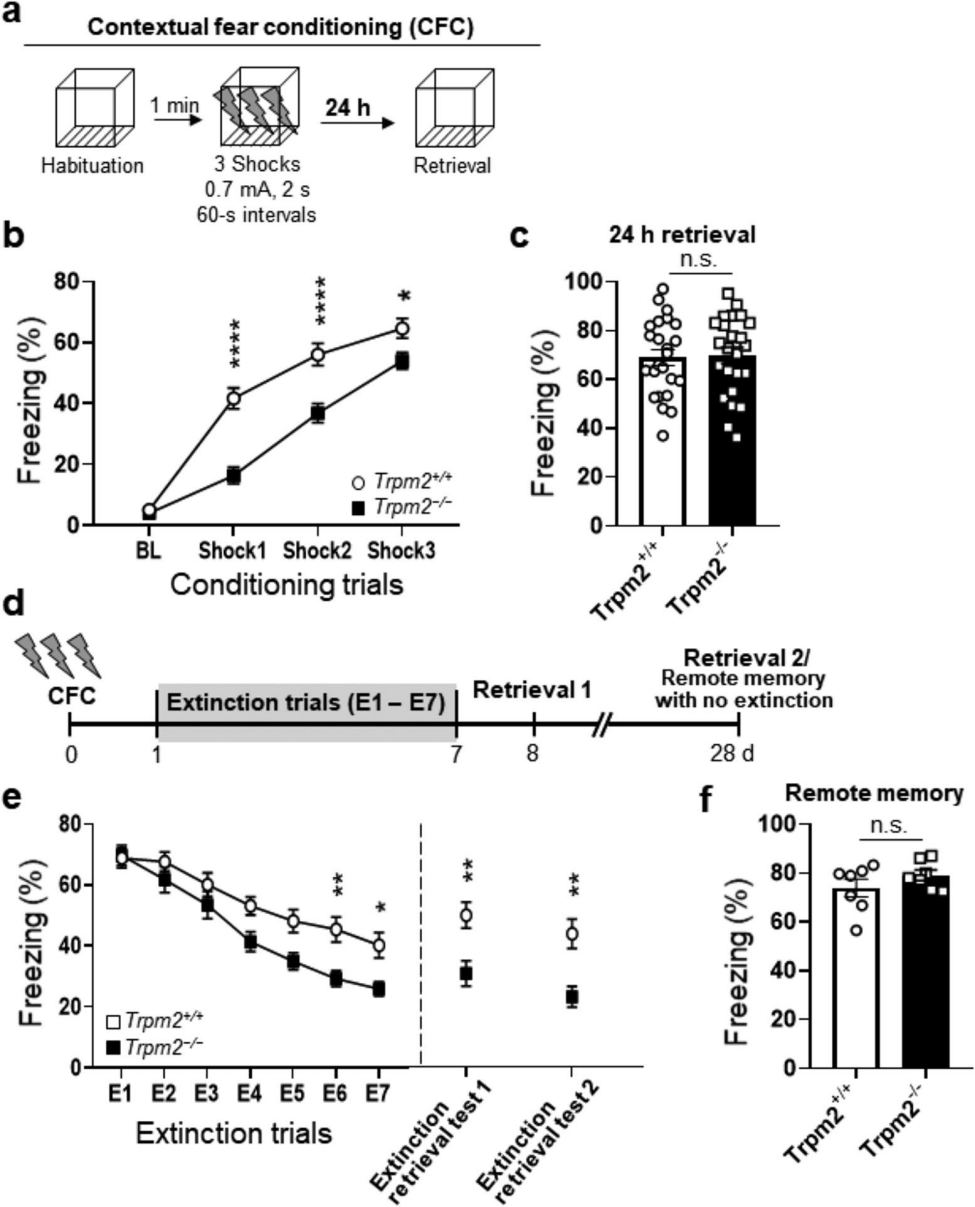
Facilitated extinction of contextual fear memory in *Trpm2*^**−/−**^ (**a**) Time line of the contextual fear conditioning procedure. The freezing response during habituation (BL) and acquisition was analyzed for 1 min per trial. A foot shock (2-sec, 0.7 mA) was given at the end of habituation and the first three conditioning trials. (**b**) *Trpm2*^*−/−*^ mice displayed reduced contextual fear acquisition (two-way repeated measures ANOVA, genotype: F_(1,44)_ = 23.53, *p* < 0.0001; shock: F_(3,132)_ = 248.3, *p* < 0.0001; genotype × shock interaction: Interaction, F_(3,132)_ = 12.26, *p* < 0.0001; *Bonferroni post hoc*, 1st, *p* < 0.0001; 2nd, *p* < 0.0001; 3rd, *p* = 0.0276; WT, *n* = 23; *Trpm2*^*−/−*^, *n* = 23). (**c**) Similar levels of freezing during fear retrieval 24 h after CFC and the first 5 min of extinction training session E1 (unpaired two-tailed t test, genotype: t_(46*)*_ = 0.1876, *p* = 0.852; WT, *n* = 24; *Trpm2*^*−/−*^, *n* = 24). (**d**) Time line of the contextual fear extinction procedure. During the extinction phase, the mice were placed in the chamber for 5 min without reinforcing shock. 24 h later, consolidated extinction memory was recalled by monitoring freezing behavior for 2 min in the original chamber. (**e**) *Trpm2*^*−/−*^ mice showed a faster rate of contextual fear extinction over the 7-day course of extinction training (two-way repeated measures ANOVA, genotype: F_(1,46)_ = 6.369, *p* = 0.0151; day: F_(6,276)_ = 65.95, *p* < 0.0001; genotype × day interaction: Interaction F_(6,276)_ = 3.067, *p* = 0.0064; *Bonferroni post hoc*, E2, *p* > 0.999; E3, *p* > 0.999; E4, *p* = 0.1266; E5, *p* = 0.0556; E6, *p* = 0.0085; E7, *p* = 0.0278; WT, *n* = 24; *Trpm2*^*−/−*^, *n* = 24). Extinction retrieval tests 24 h (at 8 d: retrieval 1) and 21 d (at 28 d: retrieval 2) after extinction training showed that *Trpm2*^*−/−*^ mice had less context-dependent freezing behavior to the conditioning context 24 h and 21 d after extinction training than WT mice (unpaired two-tailed t test, 24 h, *p* = 0.0065, WT, *n* = 8; *Trpm2*^*−/−*^, *n* = 8; 28 d, genotype: t_(23)_ = 3.535, *p* = 0.0018, WT, *n* = 12; *Trpm2*^*−/−*^, *n* = 13). (**f**) Remote memory. Conditioned mice without extinction training were returned to the context 28 d later for the remote memory test. There were no significant differences in the percentage durations of freezing between WT and *Trpm2*^*−/−*^ mice at day 28 (*p =* 0.2405). Animal freezing is measured as percent time spent freezing over a given test period. *p < 0.05, **p < 0.01, ***p < 0.001, ****p < 0.0001 compared with WT littermates. Numbers in parentheses denote the number of mice in each group used for the experiment. All data are mean ± SEM. Detailed statistics in Supplementary Information

Mice were handled for 5 consecutive days prior to the commencement of contextual fear conditioning. They were trained and tested in conditioning chambers (17.5 × 17.5 × 15 cm) that had a stainless steel grid floor through which a foot shock could be delivered [15]. Mice were first acclimated to the conditioning chamber for 3 min, which were used for baseline (BL) to determine basal freezing behavior. Next, a footshock (0.7 mA, 2 s duration) was delivered three times at 60-s intervals. Mice were returned to their home cage 1 min after the shocks (training). After the final shock, a 1 min post-paring session was used to determine fear memory acquisition (Fig. 1a, detailed schematic). During extinction training, mice were repeatedly re-exposed to the conditioning chamber for 5 min once daily for 7 days (E1-7) without delivering a shock. A video of freezing activity was recorded during each session (Video Freeze, Med Associates) and the time spent freezing in each session (baseline, CFC, extinction) was divided by the total length of each session to generate a percentage freezing time (% time) per session.

### Open field test (OFT)

Mice were placed in a corner of a white plastic box (50 × 50 × 20 cm) to initiate the test session, and their movements were recorded for 5 min with a web camera (HD C310, Logitech, Switzerland) fixed over the apparatus. Total locomotor activity was measured using an ANY-maze video tracking system (Stoelting Co., IL, USA). To quantify the spontaneous motor activity of the animals we analyzed the traveled distance and the average speed of the animals. Also, to assess the baseline anxiety levels of the animals, we measured the time the animals spent in the center of the area. After each test, the arena was thoroughly cleaned with 70% ethanol.

### Elevated plus maze (EPM) test

The maze consisted of two white acrylic open arms (30 × 5 cm) and two closed arms (30 × 5 × 15 cm) that formed the shape of a ‘plus’ sign. The apparatus was elevated to a height of 70 cm from the floor. Mice were placed in the central square (5 × 5 cm) facing the corner between a closed arm and an open arm, and allowed to explore the elevated plus maze for 5 min. The times spent in the open arm and closed arm were analyzed as a measure of anxiety.

### Novel object recognition (NOR) test

The novel object recognition test was performed in an open field arena. During the habituation period, mice were allowed to explore the empty arena for 5 min on three consecutive days. Twenty-four hours after habituation, they were exposed to the familiar arena with two identical objects placed at an equal distance for 5 min for the training. Then, one of the objects was replaced with a new object and working memory was assessed by exposing the mice to this situation for 5 min and recording time spent exploring the new *versus* the old object 100 min (short-term memory) and 24 h (long-term memory) after the training [16].

### Stereotaxic surgery

Stereotaxic surgery was performed as previously described [2]. Mice were anesthetized with an intramuscular injection of a cocktail containing ketamine 60 mg/ml, acepromazine 1 mg/ml and xylazine 8 mg/ml (0.1 ml/kg, intramuscular), and mounted onto a stereotaxic frame. For intra-DG microinjections, WT mice received a bilateral guide cannula (22-gauge; Plastics One, Roanoke, VA, USA) targeting the DG (coordinates: anteroposterior (AP) = − 2.0 mm, mediolateral (ML) = ± 1.4 mm, dorsoventral (DV) = − 2.2 mm from the bregma) as previously described [2]. Following surgery, they were individually housed, handled daily and allowed to recover for 7 days. Intra-DG microinjections were performed on conscious, unrestrained and freely moving mice in their home cage. On the experimental day, a 28-G stainless-steel injector connected to a 5-µl syringe was inserted into the guide cannula and extended 0.5 mm beyond the tip. FFA (50.6 µg/µl) or artificial cerebrospinal fluid was infused bilaterally in a volume of 4.0 µl (2.0 µl per side) over 5 min. The injector tips were held in place for an additional 5 min after the end of infusion to avoid backflow.

### Drugs

Flufenamic acid (FFA; Sigma, MO, USA) was dissolved in 80% EtOH, and FFA or vehicle (80% EtOH) was injected intra-DG 1 h before the testing session. FFA inhibits the voltage-gated sodium currents of TRPM2 in hippocampal pyramidal neurons (IC50, 189 µM) [17]. The FFA was diluted in saline to a final EtOH concentration of 1%.

### Hippocampal dissection

Mice were sacrificed after the probe trial by cervical dislocation, and their brains were removed from the skull and chilled in ice-cold HBSS All further manipulations were performed on an ice-cooled plate. Whole hippocampus was dissected from the brains, and 500 μm-thick slices, transverse to the longitudinal axis, were cut with a Starrett tissue chopper. The DG was microdissected by hand under a dissecting microscope. Subregional boundaries were clearly visible under these conditions. Tissues were collected and stored at − 80 °C.

### Genotyping and RT-PCR

To determine mouse genotypes, genomic DNA was isolated from tail tissue using a LaboPass™ Tissue Genomic DNA Mini Prep Kit (Cosmo Genetech, Korea). PCR was carried out using the purified genomic DNA with the primer sets PTRPM2-13F, PTRPM2-10R, and Pneo-5’a; their sequences were PTRPM2-13F: 5’-CTTGGGTTGCAGTCATATGCAGGC-3’, PTRPM2-10R: 5’- GCCCTCACCATCCGCTTCACGATG-3’, and Pneo5’a: 5’-GCCACACGCGTCAC CTTAATATGCG-3’.

### Quantitative real-time PCR

Total RNA was isolated from mouse hippocampal tissue using Trizol reagent (Sigma). Reverse transcription of 1 µg of total RNA was performed with oligo-dT primers using an Improm-II™ Reverse Transcription System (Promega). The resulting cDNA was used as template to amplify target gene transcripts by real time PCR. Quantitative real-time PCR (qPCR) was performed on a CFX96 Touch™ Real-Time PCR Detection System (BioRad Laboratories, CA, USA) using SensiFAST™ SYBR No-ROX mix (Bioline) according to the instructions of the manufacturer. PCR primer sequences were *c-Fos*, F: 5’-TCACCGTGGGGATAAAGTTG-3’, R: 5’-CCGACTCCTTCTCCAGCAT-3’; *Arc*, F: 5’-TACCCCTCATCTGTCTGCC-3’, R: 5’-GCCTACTTTTTGTTGCCTTTC-3’; *Egr1*, F: 5’-GACGAGTTATCCCAGCCAAA-3’, R: 5’-GGCAGAGGAAGACGATGAAG-3’; *Npas4*, F: 5’-AGCATTCCAGGCTCATCTGAA-3’, R: 5’-GGCGAAGTAAGTCTTAGGATT-3’; *β-Actin*, F: 5’-AAGGCCAACCGTGAAAAGAT-3’, R: 5’-GTGGTACGACCAGAGGCATAC-3’. All gene expression values were normalized to those of *β-actin*.

### Statistical analysis

Statistical analysis was performed with GraphPad Prism 8.0 software. Statistical differences between two groups were analyzed using unpaired two-tailed t-tests, while differences between two groups at different time points or sessions were analyzed using two-way repeated measures ANOVA. *p* < 0.05 was considered statistically significant. All experiments were carried out at least three times. Relevant statistical parameters are specified in the figure legend. Sample sizes were determined based on similar experiments carried-out in the past and in the literature. For behavioral experiments the investigators were blind to group allocation during data collection and analysis. All behavioral sessions were video recorded, and an experimenter blinded to group identity performed the manual scoring to determine freezing behavior. The exact sample size is given in each figure legend, and also shown by the individual dots in the figures. All plotted data are mean ± SEM. Relevant statistical parameters are presented in Table S1 of Supplementary Information.

## Results

### Fear extinction is facilitated in *Trpm2*^*−/−*^ mice

We used the fear conditioning paradigm to assess the behavioral responses of *Trpm2* deficient (*Trpm2*^*−/−*^) mice to contextual cues (Fig. 1a). WT and *Trpm2*^*−/−*^ littermates were trained to associate an unconditioned stimulus (3 foot shocks; 0.7 mA, 2 s duration, 60 s inter-shock interval) with a context (the shock chamber). Freezing responses did not differ between genotypes in the absence of the unconditioned stimulus, as seen at baseline (BL) (Fig. 1b). During contextual fear-conditioning (CFC) training, *Trpm2*^*−/−*^ mice froze significantly less than WT mice in all 3 trials (Fig. 1b), suggesting that acquisition of fear memory was altered in *Trpm2*^*−/−*^ mice. When we measured freezing responses 24 h after CFC in the same apparatus that was used for the conditioning in order to see long-term memory (LTM), there was no significant difference in freezing levels between WT and *Trpm2*^*−/−*^ mice (Fig. 1c). These results indicate that although the acquisition of fear memory is in part suppressed in *Trpm2*^*−/−*^ mice (on the conditioning day), *Trpm2*^*−/−*^ mice display normal contextual freezing in LTM tests compared with WT counterparts.

Twenty-four hours after conditioning, the mice received extinction training for 7 days by daily re-exposure to the conditioning context without foot shock (Fig. 1d). As expected from the results in Fig. 1c, freezing responses during extinction training session E1 did not differ between WT and *Trpm2*^*−/−*^ mice (Fig. 1e). But on subsequent days the mutant mice displayed significantly lower freezing levels than WT mice (E1–7) (Fig. 1e). *Trpm2*^*−/−*^ mice also had a significantly shorter freezing time in context-induced freezing responses during retrieval tests 24 h after extinction training (Fig. 1e, retrieval test 1 at 8 d), indicating that *Trpm2* deficiency facilitates extinction of contextual fear. Moreover, freezing responses remained lower in *Trpm2*^*−/−*^ mice than in WT mice 3 wks after extinction (Fig. 1e, retrieval test 2 at 28 d). Conditioned mice without extinction training were returned to the context 28 d later for the remote memory test. There were no significant differences in the percentage durations of freezing between WT and *Trpm2*^*−/−*^ mice at day 28, indicating that there were no deficits in remote memory in the *Trpm2*^*−/−*^ mice with intact remote memory (Fig. 1f), Together, these results indicate that fear extinction (FE) had occurred and was long-lasting.

**Fig. 2:**
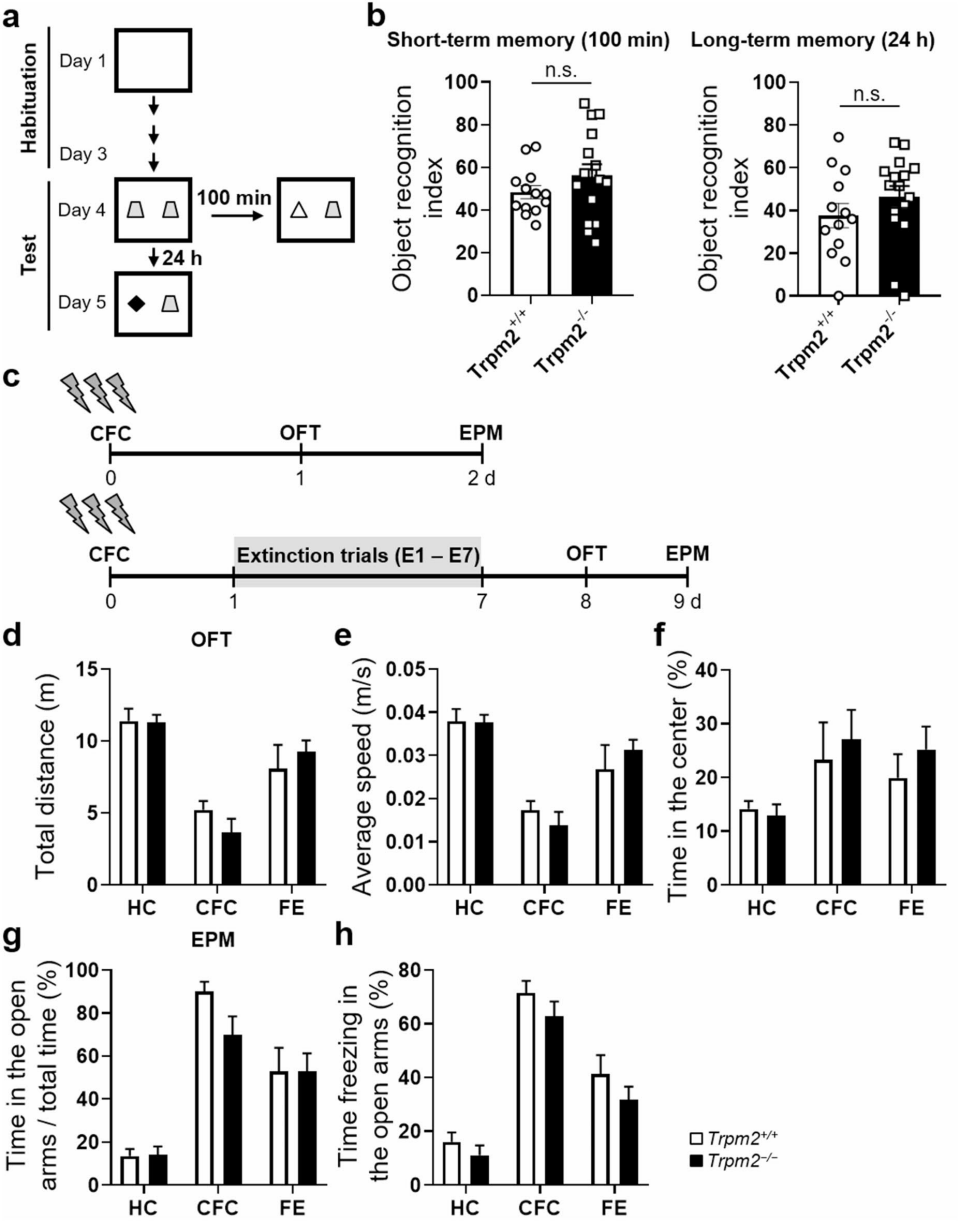
Behavioral tests related to hippocampus-dependent learning and anxiety in *Trpm2*^**−/−**^**mice.** (**a**) Experimental procedure for the novel object recognition (NOR) test. (**b**) NOR test. *Trpm2*^*−/−*^ mice had the same discrimination index as WT mice 100 min and 24 h after training (2 h, t_(27)_ = 1.265, *p* = 0.2165; 24 h, t_(27)_ = 1.149, *p* = 0.2607; WT, *n* = 13; *Trpm2*^*−/−*^, *n* = 16). (**c**) Time line of the CFC and extinction procedure followed in the OFT (**d, e, f**) and the EPM (**g, h**). Spontaneous motor activity and anxiety levels are not changed in *Trpm2*^*−/−*^ mice. Spontaneous motor activity as indicated by total distance moved and speed of mice were also not changed in the *Trpm2*^*−/−*^ mice (**d, e**; detailed statistics in Supplementary Information). Also, anxiety was not altered in the *Trpm2*^*−/−*^ mice, as there were no differences in the time animals spent in the central zone of the arena in the OFT (**f**), the number of open arm entries/total entries (**g**) and the time spent freezing in the open arm (**h**) in the EPM for 5 min (detailed statistics in Supplementary Information). Data are expressed as mean ± SEM

In order to assess whether other forms of hippocampus-dependent learning were also affected, we conducted the novel object recognition (NOR) test (Fig. 2a). Discrimination indexes 100 min and 24 h after training did not differ between WT and *Trpm2*^*−/−*^ mice (Fig. 2b), confirming that *Trpm2*^*−/−*^ is not defective in memory formation, consolidation and retention. We previously showed that *Trpm2* deletion did not affect basal locomotor activity in an open field test (OFT) [2]. *Trpm2*^*−/−*^ mice did not show any changes in spontaneous motor activity compared with WT mice after CFC and FE in any quantified activity-related behaviors in the OFT (traveled distance and average speed) (Fig. 2d, e). To assess the anxiety of the animals, we compared the times they spent in the center of the arena and found there were no differences between WT and *Trpm2*^*−/−*^ mice (Fig. 2f), indicating that anxiety behavior was unchanged in the *Trpm2*^*−/−*^ mice. The anxiety behaviors were analyzed in more detail using the elevated plus maze test (EPM), and *post-hoc* comparisons revealed no significant differences between the two genotypes in the proportion of open arm entries (Fig. 2g). Both WT and *Trpm2*^*−/−*^ mice remained freezing most of the time in the open arm (Fig. 2h). These results suggest that the enhanced extinction observed following the extinction trials was not due to abnormalities of spontaneous locomotor activity or of anxiety, but rather appeared to be related to memory processes. Taken together, these results indicate that *Trpm2* deficiency leads to impaired acquisition but intact retrieval of contextual fear memory, and enhanced extinction of contextual fear memory. Although contextual fear memories appear to be slightly impaired during conditioning, this subtle disparity was not sufficient to cause obvious abnormalities in hippocampus-related LTM.

### Expression of IEG mRNAs is downregulated in *Trpm2*^*−/−*^ mice after extinction

**Fig. 3:**
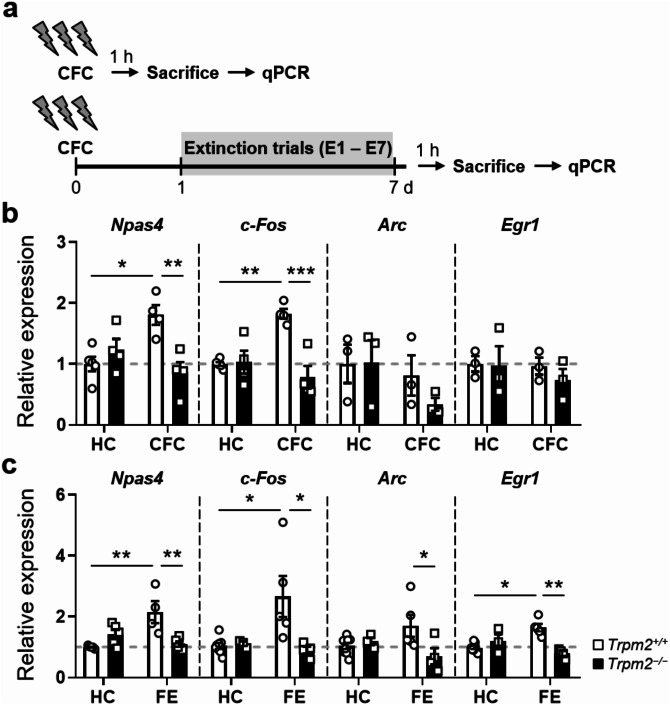
Differential expression of IEGs in the hippocampus of ***Trpm2***^**−/−**^**mice** after contextual fear conditioning and fear extinction. (**a**) The hippocampus was dissected from mice 1 h after CFC, 1 h after E7, or from mice removed from their home cage (HC). (**b**) mRNA levels of *Npas4, c-Fos, Arc* and *Egr1* in mice that underwent CFC (detailed statistics in Supplementary Information). (**c**) *Npas4*,* c-Fos* and *Egr1* *mRNA* increased in the hippocampus after extinction in WT mice but not in *Trpm2*^*−/−*^ mice. *Trpm2*^*−/−*^ mice contained less *Npas4, c-Fos, Arc* and *Egr1 mRNA* than WT mice after FE (day 8). (detailed statistics in Supplementary Information). Relative levels of IEG mRNAs measured in the hippocampus 1 h after contextual fear conditioning. Results for gene of interest mRNAs were normalized with the level of β-Actin. The mRNA levels during CFC and FE are shown as fold changes relative to the levels in the home cage WT. Values represent mean ± SEM (*n* = 3 to 6 per group). * *p* < 0.05, ** *p* < 0.01, *** *p* < 0.001. Error bars represent mean ± SEM

Given that the presentation of CS and US triggers strong activation of IEG mRNAs in hippocampal neurons during fear conditioning [18], we focused on the following: (1) the expression of IEGs mRNA in the hippocampus induced by contextual fear conditioning and extinction; and (2) the regulation of this expression by the TRPM2. We first examined whether *Trpm2* deficiency influences IEGs expression during fear conditioning in the hippocampus. The mice used were trained in parallel with the mice that were used for the behavior testing. Control animals were exposed to the home cage without CFC. To assess neuronal activation in hippocampus-dependent contextual fear memory, dissected the DG from the mice 1 h after CFC and subjected them to quantitative PCR. The IEG genes *Arc, c-Fos*, and *Npas4* were prime choices because of their well-known roles in regulating fear-related learning and memory [19, 20, 21]. We also included *Egr1*, as it is highly expressed in the brain, and induced during neural activity [22, 23]. An analysis of learning-induced IEG mRNA expression revealed that *Npas4* and *c-Fos*, but not *Arc* and *Egr1*, increased significantly compared with their home cage levels in the WT, but not *Trpm2*^*−/−*^, mice in response to CFC (Fig. 3b). As a result, there were significant differences in the expression of *Npas4* and *c-Fos* between the two genotypes 1 h after CFC (Fig. 3b). Levels of *Arc* and *Egr1* remained at baseline 1 h after learning in the animals that underwent CFC.

We next determined the effect of fear extinction on the expression of IEGs. Figure 3c shows that *Npas4*,* c-Fos*, and *Egr1* mRNAs were elevated in the WT but not *Trpm2*^*−/−*^ mice 1 h after extinction (Fig. 3c). This resulted in significantly lower expressions of *Npas4*,* c-Fos*, and *Egr1* mRNAs in *Trpm2*^*−/−*^ mice than in WT mice. Expression of *Arc* in *Trpm2*^*−/−*^ mice is below basal levels seen in home-caged WT mice. Together, these results suggest that *Trpm2* deficiency attenuates IEG induction during FE.

### Administration of the TRPM2 inhibitor FFA during the extinction trials promotes fear extinction in WT mice

**Fig. 4:**
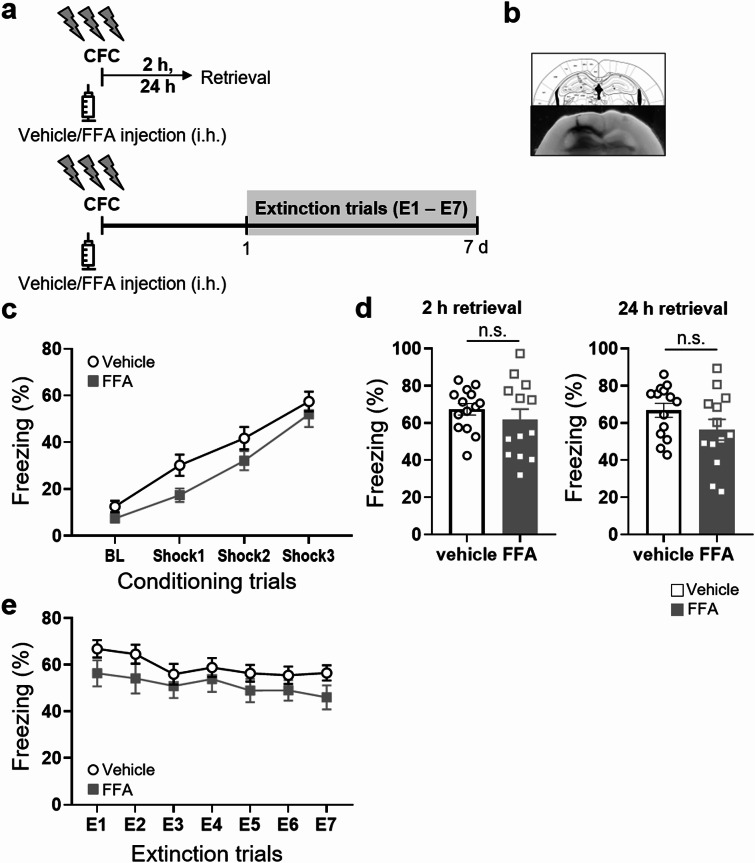
Lack of effect of intra-DG infusion of FFA before CFC on fear extinction in WT mice. (**a**) Time line of FFA infusion before the CFC procedure. (**b**) Microinjection sites in the DG (closed circles for FFA-treated mice). (**c-d**) Dentate gyrus (DG)-cannulated WT mice received intra-DG infusion of FFA or vehicle 1 h before CFC, followed by CFC and extinction. In fear-conditioned mice, intra-DG infusion of FFA did not affect freezing responses during conditioning training (two-way repeated measures ANOVA, drug, F_(1,25)_ = 3.772, *p* = 0.0635; shock F_(3,75)_ = 79.56, *p* < 0.0001; Drug x Shock Interaction, F_(3,75)_ = 0.7430, *p* = 0.5298; Veh, *n* = 14, intra-DG FFA, *n* = 13). (**d**) Effects of FFA treatment on memory retrieval. Freezing responses to the context 2 h (2 h retrieval) and 24 h after CFC (24 h retrieval). Different sets of mice received micro-infusion of FFA or Veh into the dorsal hippocampus 1 h before CFC. After conditioning, the FFA-treated mice displayed similar freezing responses over 5 min in the conditioning context compared with vehicle-treated mice (2 h, t_(25)_ = 0.8843, *p* = 0.385; Veh, *n* = 14; intra-DG FFA, *n* = 13; 24 h, (t_(25)_ = 1.589, *p* = 0.1246; Veh, *n* = 14; intra-DG FFA, *n* = 13). Freezing was assessed as percentage of time spent freezing in the training context. (**e**) FFA did not affect freezing responses during E1-7 (two-way repeated measures ANOVA, treatment: F_(1,25)_ = 2.371, *p* = 0.1362; day: F_(6,150)_ = 3.248, *p* = 0.05; treatment × day interaction F_(6,150)_ = 0.3375, *p* = 0.9162; Veh, *n* = 14; intra-DG FFA, *n* = 13). Error bars represent mean ± SEM

Given the abundant expression of TRPM2 in the DG of the hippocampus [12] and the critical role of this brain region in contextual FE [24, 25, 26], we reasoned that the effects of *Trpm2* deletion could be a key factor in mediating facilitation of contextual FE in the hippocampus. To test this possibility, mice were bilaterally cannulated in the DG and subjected to same version of the fear conditioning and extinction paradigm (Fig. 4a, b) in the presence and absence of flufenamic acid (FFA). FFA is an effective antagonist of human TRPM2 channels and ADP-ribose activated currents in rodents [27]. FFA (200 µM) given 1 h before conditioning did not alter freezing responses during the conditioning session (Fig. 4c). To ensure that the FFA did not weaken retrieval and retention of the fear memory, we measured freezing responses to context in a drug-free state 2 h and 24 h after fear conditioning, in a separate set of animals (Fig. 4d), and found that freezing levels did not differ between vehicle and FFA-treated mice either 2–24 h after fear conditioning (Fig. 4d). These results confirm that FFA did not cause deficits in the acquisition, retrieval or retention of fear memory. During the extinction training, freezing to the conditioned context did not differ significantly across the extinction sessions in either group (Fig. 4e).

**Fig. 5:**
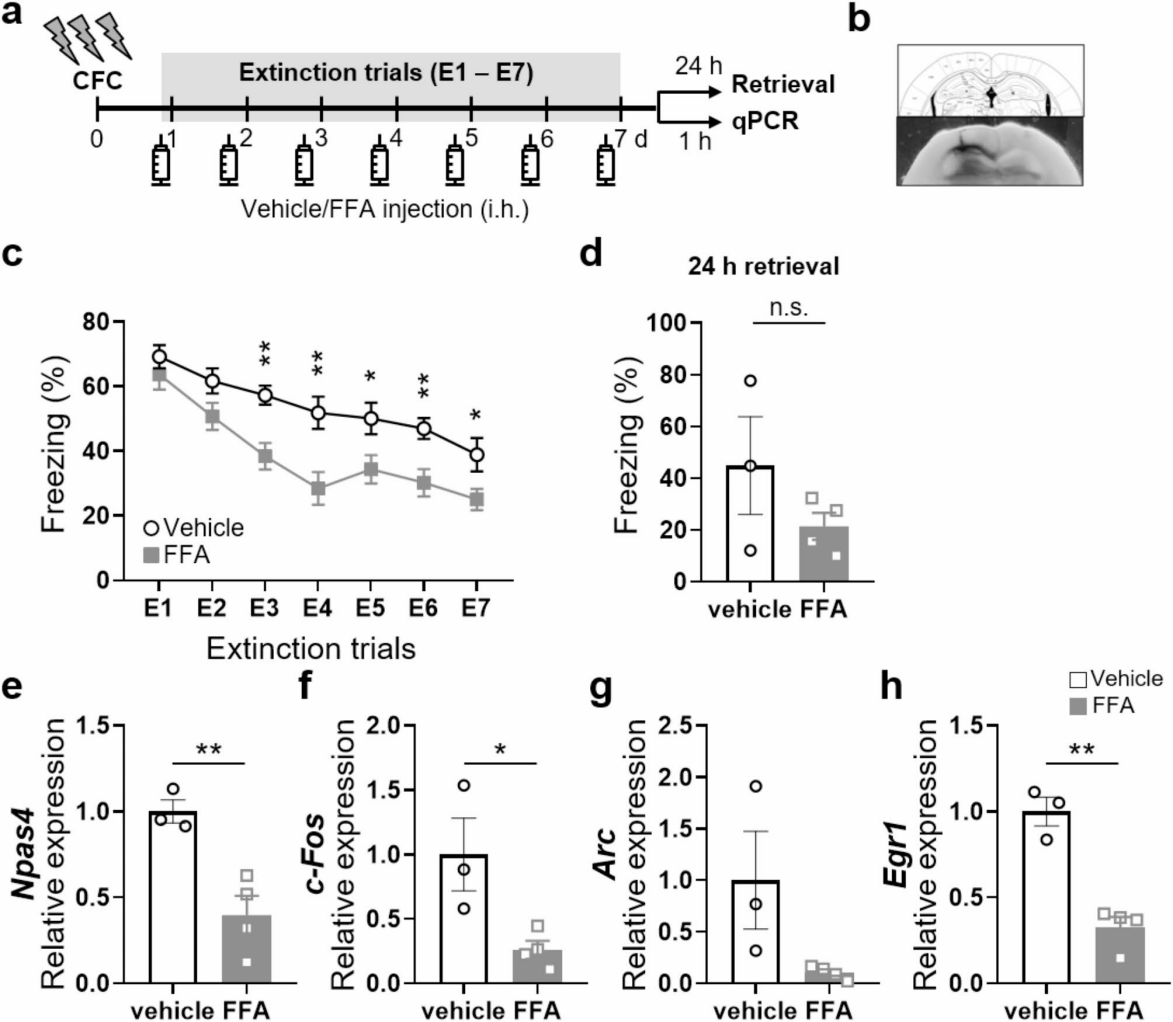
Facilitation of contextual fear extinction by intra-DG infusions of FFA during extinction. (**a**) Time line of FFA infusion during extinction procedure. Dentate gyrus (DG)-cannulated WT mice received intra-DG infusions of FFA or vehicle during extinction training over 7 days (E1–7). Intra-DG infusion was given daily 1 h before re-exposure to the context during extinction training. (**b**) Microinjection sites in the DG. (**c**) The FFA group froze significantly less than the Veh group during the extinction training, especially during E2-7 (Unpaired two-tailed t test, E2, t_(21)_ = 1.912, *p* = 0.0696; E3, t_(21)_ = 3.695, *p* = 0.0013; E4, t_(21)_ = 3.289, *p* = 0.0035; E5, t_(21)_ = 2.399, *p* = 0.0258; E6, t_(21)_ = 3.119, *p* = 0.0052; E7, t_(21)_ = 2.300, *p* = 0.0318; see also Supplementary Information). Overall, mice that received intra-DG infusions of FFA spent less time in freezing during extinction sessions 1 to 7 (two-way ANOVA, drug: F_(1,21)_ = 9.451, *p* = 0.0058; extinction: F_(6,126)_ = 38.44, *p* < 0.0001; interaction, drug x extinction: F_(6,126)_ = 2.281, *p* = 0.04; Veh, *n* = 11; intra-DG FFA, *n* = 12). (**d**) Results of extinction retrieval tests 24 h after the extinction training. There was no difference in the freezing response between the Veh- and FFA-treated groups (*p* > 0.1). (**e-h**) Downregulation of IEG mRNAs by intra-DG infusions of FFA during extinction. The expression of IEG mRNAs in each group is presented as the ratio in the FFA group relative to the Veh group (Unpaired two-tailed t test, *Npas4*, t_(5)_ = 4.206, *p* = 0.0084; *c-Fos*, t_(5)_ = 2.959, *p* = 0.0315; *Arc*, t_(5)_ = 2.246, *p* = 0.0746; *Egr1*, t_(5)_ = 6.762, *p* = 0.011; Veh, *n* = 3; intra-DG FFA, *n* = 4. *p < 0.05, **p < 0.01, compared with the vehicle-treated group). Error bars represent mean ± SEM

Finally, fear-conditioned mice received intra-DG infusion on the day before extinction training, and over the course of the 7-day extinction training FFA was infused into the DG 1 h before each extinction session (Fig. 5a). Overall, fear-extinction was accelerated in the mice that received FFA: they spent less time freezing during the extinction sessions than the vehicle-treated control mice (Fig. 5c). However, twenty-four hours later, tested in the conditioning context in a drug-free state, the mice given FFA before the extinction training exhibited similar levels of freezing to the control mice (Fig. 5d). To see whether FFA treatment also downregulated IEG expression, animals were treated with FFA or vehicle, and levels of IEG mRNAs were measured in the DG 1 h after extinction training. Expression of *Npas4, c-Fos* and *Egr1*, and especially of *Arc* was lower in the FFA-treated mice than the control mice (Fig. 5e, f, g, h).

Thus, collectively, our observations of the effects of *Trpm2* deletion and FFA administration concur in indicating that TRPM2 activity contributes to inhibition of the extinction of contextual fear memory.

## Discussion

In this study, we demonstrate, for the first time, that TRPM2 plays an important role in inhibiting the extinction of contextual fear memory. We found that mice deficient in *Trpm2* exhibited enhanced extinction learning. This is unlikely to be due to developmental compensation/adaptation in the *Trpm2*^*−/−*^ mice since WT mice receiving infusions of FFA into the DG directly before extinction training also exhibited facilitated extinction of contextual fear. The facilitated extinction of contextual fear in *Trpm2*^*−/−*^ mice was associated with blunted expression of IEGs in the hippocampus. One obvious mechanism that would account for the facilitated extinction of fear memory is that deleting/inhibiting the TRPM2 ion channel reduces neuronal excitability and thus cell signaling. The fact that both treatments reduced the expression of IEGs, would agree with the finding that TRPM2 is involved in fear memory formation. Although further experiments are needed to address how these TRPM2 and IEGs interact at the cellular level to facilitate fear memory extinction at the cellular level, our results suggest that TRPM2 may regulate intrinsic excitability of hippocampal neurons resulting in changes in the cellular mechanisms underlying fear extinction behaviors.

It has been previously shown that *Trpm2* deficiency causes impaired LTD [7], LTP, and hippocampal-dependent memory including long-term contextual fear memory [8]. In contrast, our results show that *Trpm2* deficiency does not overtly affect hippocampus-dependent memory such as NOR, retrieval, and long-term contextual fear memory. The reason for the discrepancy with regard to LTM probably lies in the fear conditioning procedures used to explore the effects of *Trpm2* deficiency on long-term contextual fear memory. In the previous study, the authors used classical fear conditioning, while we used contextual fear conditioning. Classical fear conditioning is a more complex Pavlovian fear conditioning protocol in which auditory tones and foot shocks are presented at different times. The complexity of this protocol requires additional circuitry in the medial prefrontal cortex [28] and thus may contribute to the formation of LTM, which is regulated at multiple levels.

*Trpm2*^*−/−*^ mice are also capable of contextual fear learning but expression of the fear response is in part suppressed until re-exposure to the conditioning context. In the *Trpm2*^*−/−*^ mice, contextual memory of noxious environments might be formed but remain suppressed and capable of being retrieved subsequently as fear memory. Considering the diverse and controversial neurophysiological effects of TRPM2, it is difficult to speculate on how it might regulate extinction and IEGs expression. Further study of TRPM2-regulated events including IEG expression within the neural circuitry underlying FE may prove useful in uncovering how TRPM2 functions to regulate unwanted fearful memories. This has been the goal of many treatments for various anxiety disorders, such as PTSD, phobias, and social anxiety.

The expression of IEGs is dynamically regulated in response to neuronal activity in the brain [29, 30, 31, 32]. Our experiments show that exposure to CFC or FE conditions increases IEG expression in the WT hippocampus, and both CFC and FE resulted in lower expression of *Npas4* and *c-Fos* mRNAs in common in the *Trpm2*^*−/−*^mice than in the WT at the time points we tested. These observations suggest that TRPM2 is functionally coupled to IEGs to support hippocampus-dependent memory. However, it is possible that we missed an increase of IEGs at very early times, based on the fact that IEG expression is detected in the brain within minutes of a behavioral experience [29, 30, 33]. In summary, while TRPM2 may not be critical for the LTM of contextual fear, learning-related plasticity (as measured with IEGs) may be altered in the *Trpm2*^*−/−*^ mice, and the consolidation or retrieval of memory is likely to be attributable to this learning-induced plasticity.

Interestingly, although FFA treatment also facilitated extinction learning in WT mice, this effect was transient and did not persist 24 h after extinction training, unlike the persistent effects seen in *Trpm2*^*−/−*^ mice. This discrepancy may be due to the difference in the mechanisms of genetic deletion versus pharmacological blockade. FFA, a TRPM2 antagonist, may have a reversible effect, as previous studies have shown that the drug’s effects on ion currents can wash out after administration [17]. In contrast, *Trpm2*^*−/−*^ mice have a permanent genetic deletion of *Trpm2*, leading to more sustained effects on fear extinction. Additionally, the broad spectrum of effects that FFA has on other ion channels could contribute to the differences observed between the two approaches [34].

The findings of this study also suggest that TRPM2 and its interaction with IEGs could offer novel targets for therapeutic strategies in anxiety and trauma-related disorders, such as PTSD. Given the enhanced extinction observed in *Trpm2*^*−/−*^ mice, TRPM2 inhibition may be a promising avenue for facilitating the extinction of fear memories in patients with PTSD and other anxiety disorders. Future studies should explore how TRPM2 and IEGs interact within neural circuits to regulate the extinction of fear memories at a cellular level. It will also be important to investigate the long-lasting effects of TRPM2 inhibition and whether these effects can be harnessed for clinical therapies aimed at treating trauma- and stress-related disorders.

In conclusion, our results demonstrate that TRPM2 plays a critical role in the regulation of contextual fear extinction. *Trpm2* deficiency enhances the extinction of fear memories and reduces IEG expression, suggesting that TRPM2 modulates the intrinsic excitability of hippocampal neurons and impacts the cellular mechanisms underlying fear extinction. This research provides valuable insight into the molecular mechanisms of fear memory and offers potential therapeutic targets for anxiety disorders.

## Electronic supplementary material

Below is the link to the electronic supplementary material.

Supplementary Material 1

## Data Availability

No datasets were generated or analysed during the current study.
