# Correction: Extinction of contextual fear memory is facilitated in TRPM2 knockout mice

**DOI:** 10.1186/s13041-025-01194-x

**Published:** 2025-04-15

**Authors:** Seung Yeon Ko, Do Gyeong Kim, Huiju Lee, Sung Jun Jung, Hyeon Son

**Affiliations:** 1 Hanyang Biomedical Research Institute, Hanyang University, Seongdong-gu, Seoul, 04763 Korea; 2 Graduate School of Biomedical Science and Engineering, Hanyang University, Seongdong-gu, Seoul, 04763 Korea; 3 Department of Physiology, College of Medicine, Hanyang University, Seongdong-gu, Seoul, 04763 Korea; 4 Department of Biochemistry and Molecular Biology, College of Medicine, Hanyang University, Seongdong-gu, Seoul, 04763 Korea; 5 College of Medicine, Hanyang University, 222 Wangsimni-ro, Seongdong-gu, Seoul, 04763 Republic of Korea

**Correction: Molecular Brain (2025) 18:16** 10.1186/s13041-025-01181-2

Following publication of the original article [1], the authors identified an error in Fig. 1. Due to an error Fig. 5 was indicated also as Fig. 1. The correct figure and caption is given below.

The incorrect Fig. 1:

**Fig. 1:**
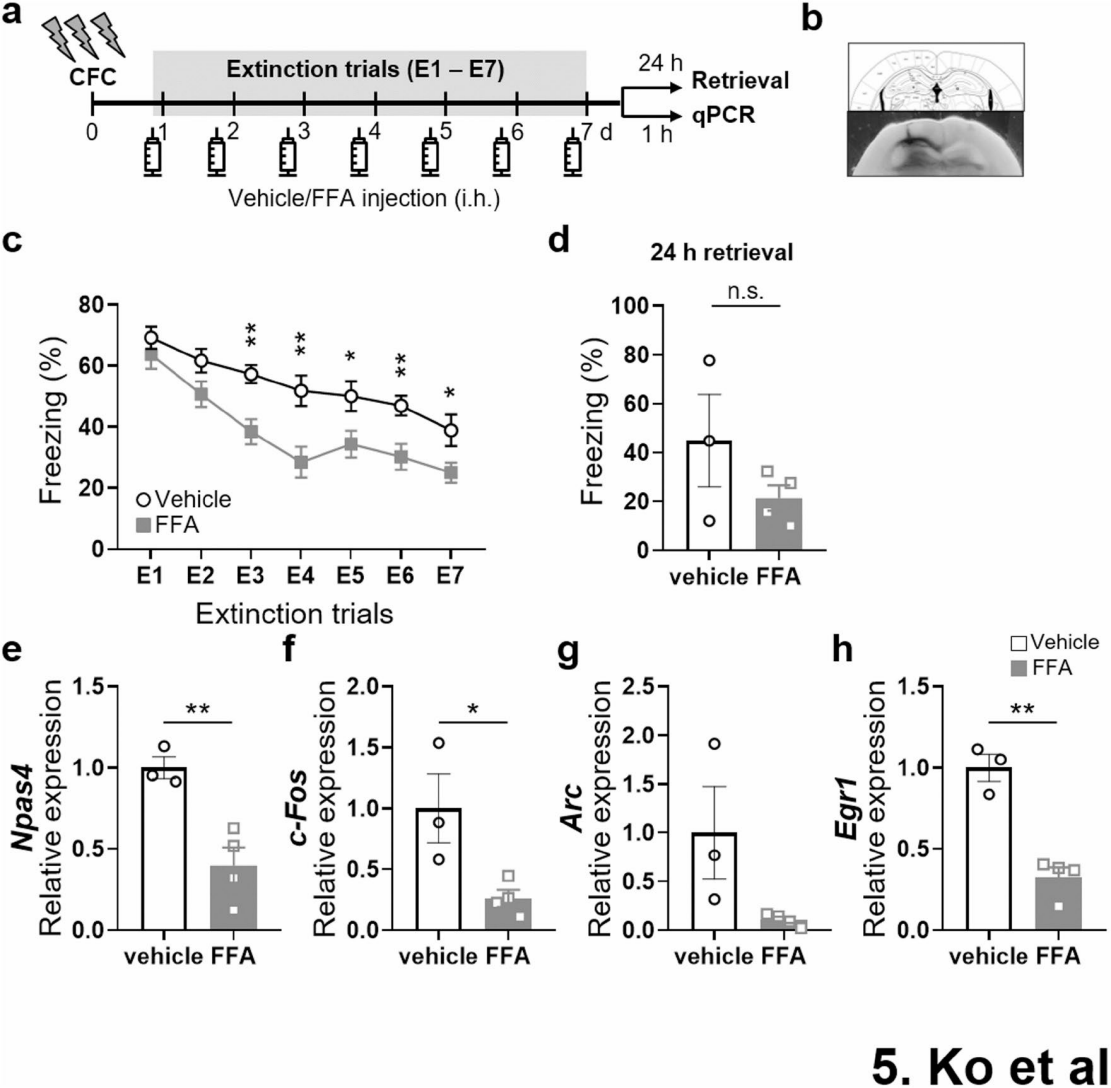
Facilitated extinction of contextual fear memory in *Trpm2*^−/−^mice. (**a**) Time line of the contextual fear conditioning procedure. The freezing re-sponse during habituation (BL) and acquisition was analyzed for 1 min per trial. A foot shock (2-s, 0.7 mA) was given at the end of habituation and the first three conditioning trials. (**b**) *Trpm2*^*−/−*^ mice displayed reduced contextual fear acquisition (two-way repeated measures ANOVA, genotype: F(1,44) = 23.53, *p* < 0.0001; shock: F(3,132) = 248.3, *p* < 0.0001; genotype × shock interaction: Interaction, F(3,132) = 12.26, *p* < 0.0001; *Bonferroni *post hoc, 1st, *p* < 0.0001; 2nd, *p* < 0.0001; 3rd, *p* = 0.0276; WT, *n* = 23; *Trpm2*^*−/−*^, *n* = 23). (**c**) Similar levels of freezing during fear retrieval 24 h after CFC and the first 5 min of extinction training session E1 (unpaired two-tailed t test, genotype: t(46*)* = 0.1876, *p* = 0.852; WT, *n* = 24; *Trpm2*^*−/−*^, *n* = 24). (**d**) Time line of the contextual fear extinction procedure. During the extinction phase, the mice were placed in the chamber for 5 min without reinforcing shock. 24 h later, consolidated extinction memory was recalled by monitoring freezing behavior for 2 min in the original chamber. (**e**) *Trpm2*^*−/−*^ mice showed a faster rate of contextual fear extinction over the 7-day course of extinction training (two-way repeated measures ANOVA, genotype: F(1,46) = 6.369, *p* = 0.0151; day: F(6,276) = 65.95, *p* < 0.0001; genotype × day interaction: Interaction F(6,276) = 3.067, *p* = 0.0064; *Bonferroni *post hoc, E2, *p* > 0.999; E3, *p* > 0.999; E4, *p* = 0.1266; E5, *p* = 0.0556; E6, *p* = 0.0085; E7, *p* = 0.0278; WT, *n* = 24; *Trpm2*^*−/−*^, *n* = 24). Extinction retrieval tests 24 h (at 8 d: retrieval 1) and 21 d (at 28 d: retrieval 2) after extinction training showed that *Trpm2*^*−/−*^ mice had less context-dependent freezing behavior to the conditioning context 24 h and 21 d after extinction training than WT mice (unpaired two-tailed t test, 24 h, *p* = 0.0065, WT, *n* = 8; *Trpm2*^*−/−*^, *n* = 8; 28 d, genotype: t(23) = 3.535, *p* = 0.0018, WT, *n* = 12; *Trpm2*^*−/−*^, *n* = 13). (**f**) Remote memory. Conditioned mice without extinction training were returned to the context 28 d later for the remote memory test. There were no significant differences in the percentage durations of freezing between WT and *Trpm2*^*−/−*^ mice at day 28 (*p* = 0.2405). Animal freezing is measured as percent time spent freezing over a given test period. *p < 0.05, **p < 0.01, ***p < 0.001, ****p < 0.0001 compared with WT littermates. Numbers in parentheses denote the number of mice in each group used for the experiment. All data are mean ± SEM. Detailed statistics in Supplementary Information

The correct Fig. 1:

**Fig. 1:**
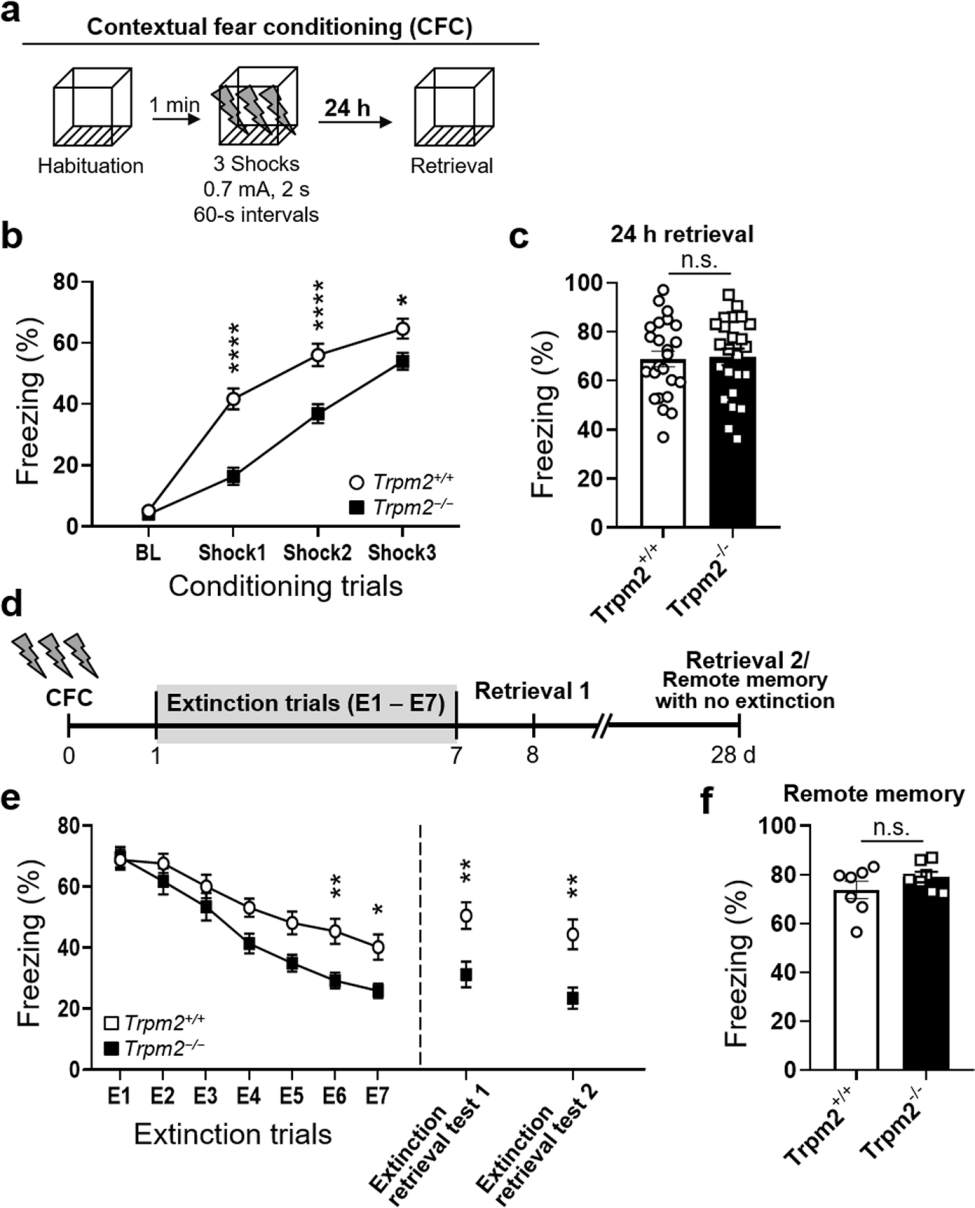
Facilitated extinction of contextual fear memory in *Trpm2*^−/−^mice. (**a**) Time line of the contextual fear conditioning procedure. The freezing re-sponse during habituation (BL) and acquisition was analyzed for 1 min per trial. A foot shock (2-s, 0.7 mA) was given at the end of habituation and the first three conditioning trials. (**b**) *Trpm2*^*−/−*^ mice displayed reduced contextual fear acquisition (two-way repeated measures ANOVA, genotype: F(1,44) = 23.53, *p* < 0.0001; shock: F(3,132) = 248.3, *p* < 0.0001; genotype × shock interaction: Interaction, F(3,132) = 12.26, *p* < 0.0001; *Bonferroni *post hoc, 1st, *p* < 0.0001; 2nd, *p* < 0.0001; 3rd, *p* = 0.0276; WT, *n* = 23; *Trpm2*^*−/−*^, *n* = 23). (**c**) Similar levels of freezing during fear retrieval 24 h after CFC and the first 5 min of extinction training session E1 (unpaired two-tailed t test, genotype: t(46*)* = 0.1876, *p* = 0.852; WT, *n* = 24; *Trpm2*^*−/−*^, *n* = 24). (**d**) Time line of the contextual fear extinction procedure. During the extinction phase, the mice were placed in the chamber for 5 min without reinforcing shock. 24 h later, consolidated extinction memory was recalled by monitoring freezing behavior for 2 min in the original chamber. (**e**) *Trpm2*^*−/−*^ mice showed a faster rate of contextual fear extinction over the 7-day course of extinction training (two-way repeated measures ANOVA, genotype: F(1,46) = 6.369, *p* = 0.0151; day: F(6,276) = 65.95, *p* < 0.0001; genotype × day interaction: Interaction F(6,276) = 3.067, *p* = 0.0064; *Bonferroni *post hoc, E2, *p* > 0.999; E3, *p* > 0.999; E4, *p* = 0.1266; E5, *p* = 0.0556; E6, *p* = 0.0085; E7, *p* = 0.0278; WT, *n* = 24; *Trpm2*^*−/−*^, *n* = 24). Extinction retrieval tests 24 h (at 8 d: retrieval 1) and 21 d (at 28 d: retrieval 2) after extinction training showed that *Trpm2*^*−/−*^ mice had less context-dependent freezing behavior to the conditioning context 24 h and 21 d after extinction training than WT mice (unpaired two-tailed t test, 24 h, *p* = 0.0065, WT, *n* = 8; *Trpm2*^*−/−*^, *n* = 8; 28 d, genotype: t(23) = 3.535, *p* = 0.0018, WT, *n* = 12; *Trpm2*^*−/−*^, *n* = 13). (**f**) Remote memory. Conditioned mice without extinction training were returned to the context 28 d later for the remote memory test. There were no significant differences in the percentage durations of freezing between WT and *Trpm2*^*−/−*^ mice at day 28 (*p* = 0.2405). Animal freezing is measured as percent time spent freezing over a given test period. *p < 0.05, **p < 0.01, ***p < 0.001, ****p < 0.0001 compared with WT littermates. Numbers in parentheses denote the number of mice in each group used for the experiment. All data are mean ± SEM. Detailed statistics in Supplementary Information

Figure 1 has been updated above and the original article [1] has been corrected.
