# Supplementary material for: Extinction of contextual fear memory is facilitated in TRPM2 knockout mice
Source: Mol Brain. 2025 Feb 27;18:16. doi: 10.1186/s13041-025-01181-2 (PMC11869647; doi:10.1186/s13041-025-01181-2)
Supplement: Supplementary file 1 — Supplementary Material 1 [file 13041_2025_1181_MOESM1_ESM.docx]

**Supplementary Information**

**Extinction of contextual fear memory is facilitated in TRPM2 knockout mice**

Seung Yeon Ko^1,†^, Do Gyeong Kim^2,†^, Huiju Lee^2,†^, Sung Jun Jung ^3,5,^*, Hyeon Son^1,4,5,^*

^1^ Hanyang Biomedical Research Institute, Hanyang University, Seongdong-gu, Seoul 04763, Korea.^2^Graduate School of Biomedical Science and Engineering, Hanyang University, Seongdong-gu, Seoul 04763, Korea. ^3^Department of Physiology, College of Medicine, Hanyang University, Seongdong-gu, Seoul 04763, Korea. ^4^Department of Biochemistry and Molecular Biology, ^5^College of Medicine, Hanyang University, Seongdong-gu, Seoul 04763, Korea.

^†^These authors contributed equally to this work

*To whom correspondence may be addressed.

Table 1. Statistical analysis

| Figure Number | Test Used | n  Exact Value | P Value | Degrees of Freedom Value |
| --- | --- | --- | --- | --- |
| 1b  CFC | Two-way RM ANOVA | *Trpm2^+/+^* (23);  *Trpm2^-/-^* (23) | Genotype x Shock  Interaction *p* < 0.0001;  Genotype *p* < 0.0001;  Shock *p* < 0.0001  Bonferroni posttest:  Baseline *Trpm2^+/+^* vs. *Trpm2^-/-^* *p* > 0.9999;  Shock1 *Trpm2^+/+^* vs. *Trpm2^-/-^* *p* < 0.0001;  Shock2 *Trpm2^+/+^* vs. *Trpm2^-/-^* *p* < 0.0001;  Shock3 *Trpm2^+/+^* vs. *Trpm2^-/-^* *p* = 0.0276 | Genotype x Shock  Interaction F_(3,132)_ = 12.26;  Genotype F_(1,44)_ = 23.53;  Shock F_(3,132)_ = 248.3 |
| 1c  24 h after CFC | Unpaired two-tailed t test | *Trpm2^+/+^* (24);  *Trpm2^-/-^* (24) | *Trpm2^+/+^* vs. *Trpm2^-/-^* *p* = 0.852 | t_(46)_ = 0.1876 |
| 1e  extinction | Two-way RM ANOVA | *Trpm2^+/+^* (24);  *Trpm2^-/-^* (24) | Genotype x Extinction  Interaction *p* = 0.0064;  Genotype *p* = 0.0151;  Extinction *p* < 0.0001  Bonferroni posttest:  E1 *Trpm2^+/+^* vs. *Trpm2^-/-^* *p* > 0.9999;  E2 *Trpm2^+/+^* vs. *Trpm2^-/-^* *p* > 0.9999;  E3 *Trpm2^+/+^* vs. *Trpm2^-/-^* *p* > 0.9999;  E4 *Trpm2^+/+^* vs. *Trpm2^-/-^* *p* = 0.1266;  E5 *Trpm2^+/+^* vs. *Trpm2^-/-^* *p* = 0.0556;  E6 *Trpm2^+/+^* vs. *Trpm2^-/-^* *p* = 0.0085;  E7 *Trpm2^+/+^* vs. *Trpm2^-/-^* *p* = 0.0278 | Genotype x Extinction  Interaction F_(6,276)_ = 3.067;  Genotype F_(1,46)_ = 6.369;  Extinction F_(6,276)_ = 65.95 |
| 1e  24 h after extinction | Unpaired two-tailed t test | *Trpm2^+/+^* (8);  *Trpm2^-/-^* (8) | *Trpm2^+/+^* vs. *Trpm2^-/-^* *p* = 0.0065 | t_(14)_ = 3.192 |
| 1e  21 after extinction | Unpaired two-tailed t test | *Trpm2^+/+^* (12);  *Trpm2^-/-^* (13) | *Trpm2^+/+^* vs. *Trpm2^-/-^* *p* = 0.0018 | t_(23)_ = 3.535 |
| 1f  Remote memory | Unpaired two-tailed t test | *Trpm2^+/+^* (7);  *Trpm2^-/-^* (7) | *Trpm2^+/+^* vs. *Trpm2^-/-^* *p* = 0.2405 | t_(12)_ = 1.235 |
| 2b  NOR 2 h | Unpaired two-tailed t test | *Trpm2^+/+^* (13);  *Trpm2^-/-^* (16) | *Trpm2^+/+^* vs. *Trpm2^-/-^* *p* = 0.2165 | t_(27)_ = 1.265 |
| 2b  NOR 24 h | Unpaired two-tailed t test | *Trpm2^+/+^* (13);  *Trpm2^-/-^* (16) | *Trpm2^+/+^* vs. *Trpm2^-/-^* *p* = 0.2607 | t_(27)_ = 1.149 |
| 2d  OFT: Total distance moved | Two-way ANOVA | *Trpm2^+/+^*  HC (16), CFC (11), FE (12);  *Trpm2^-/-^*  HC (14), CFC (10), FE (14) | Genotype x Shock  Interaction *p* = 0.3808;  Genotype *p* < 0.0001;  CFC *p* = 0.8450  Bonferroni posttest:  HC *Trpm2^+/+^* vs. *Trpm2^-/-^* *p* > 0.9999;  CFC *Trpm2^+/+^* vs. *Trpm2^-/-^* *p* > 0.9999;  FE *Trpm2^+/+^* vs. *Trpm2^-/-^* *p* > 0.9999;  HC *Trpm2^+/+^* vs. CFC *Trpm2^+/+^ p* = 0.0003;  HC *Trpm2^+/+^* vs. FE *Trpm2^+/+^* *p* = 0.1941 | Genotype x Shock  Interaction F_(2,71)_ = 0.9786;  Genotype F_(1,71)_ = 0.03849;  CFC F_(2,71)_ = 25.32 |
| 2e  OFT: Average speed | Two-way ANOVA | *Trpm2^+/+^*  HC (16), CFC (11), FE (12);  *Trpm2^-/-^*  HC (14), CFC (10), FE (14) | Genotype x Shock  Interaction p = 0.4829;  Genotype p = 0.9397;  CFC p < 0.0001  Bonferroni posttest:  HC *Trpm2^+/+^* vs. *Trpm2^-/-^* *p* > 0.9999;  CFC *Trpm2^+/+^* vs. *Trpm2^-/-^* *p* > 0.9999;  FE *Trpm2^+/+^* vs. *Trpm2^-/-^* *p* > 0.9999;  HC *Trpm2^+/+^* vs. CFC *Trpm2^+/+^* *p* = 0.0002;  HC *Trpm2^+/+^* vs. FE *Trpm2^+/+^* *p* = 0.1817 | Genotype x Shock  Interaction F_(2,71)_ = 0.7355;  Genotype F_(1,71)_ = 0.005771;  CFC F_(2,71)_ = 23.90 |
| 2f  OFT: Time spent in the central zone | Two-way ANOVA | *Trpm2^+/+^*  HC (16), CFC (11), FE (12);  *Trpm2^-/-^*  HC (14), CFC (10), FE (14) | Genotype x Shock  Interaction *p* = 0.6857;  Genotype *p* = 0.4453;  CFC *p* = 0.0133  Bonferroni posttest:  HC *Trpm2^+/+^* vs. *Trpm2^-/-^ p* > 0.9999;  CFC *Trpm2^+/+^* vs. *Trpm2^-/-^* *p* > 0.9999;  FE *Trpm2^+/+^* vs. *Trpm2^-/-^* *p* > 0.9999;  HC *Trpm2^+/+^* vs. CFC *Trpm2^+/+^* *p* > 0.9999;  HC *Trpm2^+/+^* vs. FE *Trpm2^+/+^* *p* > 0.9999 | Genotype x Shock  Interaction F_(2,71)_ = 0.3794;  Genotype F_(1,71)_ = 0.5892;  CFC F_(2,71)_ = 4.597 |
| 2g  EPM: Time in the open arms / total time | Two-way ANOVA | *Trpm2^+/+^*  HC (16), CFC (11), FE (12);  *Trpm2^-/-^*  HC (12), CFC (10), FE (14) | Genotype x Shock  Interaction *p* = 0.2805;  Genotype *p* = 0.2669;  CFC p < 0.0001  Bonferroni posttest:  HC *Trpm2^+/+^* vs. *Trpm2^-/-^* *p* > 0.9999;  CFC *Trpm2^+/+^* vs. *Trpm2^-/-^* *p* = 0.6446;  FE *Trpm2^+/+^* vs. *Trpm2^-/-^* *p* > 0.9999;  HC *Trpm2^+/+^* vs. CFC *Trpm2^+/+^* *p* < 0.0001;  HC *Trpm2^+/+^* vs. FE *Trpm2^+/+^* *p* = 0.0015 | Genotype x Shock  Interaction F_(2,69)_ = 1.295;  Genotype F_(1,69)_ = 1.253;  CFC F_(2,69)_ = 43.24 |
| 2h  EPM: Time freezing in the open arms | Two-way ANOVA | *Trpm2^+/+^*  HC (16), CFC (11), FE (12);  *Trpm2^-/^*^-^  HC (12), CFC (10), FE (14) | Genotype x Shock  Interaction *p* = 0.8754;  Genotype *p* = 0.0581;  CFC *p* < 0.0001  Bonferroni posttest:  HC *Trpm2^+/+^* vs. *Trpm2^-/-^* *p* > 0.9999;  CFC *Trpm2^+/+^* vs. *Trpm2^-/-^* *p* > 0.9999;  FE *Trpm2^+/+^* vs. *Trpm2^-/-^* *p* > 0.9999;  HC *Trpm2^+/+^* vs. CFC *Trpm2^+/+^* *p* < 0.0001;  HC *Trpm2^+/+^* vs. FE *Trpm2^+/+^* *p* = 0.2172 | Genotype x Shock  Interaction F_(2,69)_ = 0.1333;  Genotype F_(1,69)_ = 3.714;  CFC F_(2,69)_ = 58.25 |
| 3b  *Npas4* | Two-way ANOVA | *Trpm2^+/+^*  HC (5), CFC (4);  *Trpm2^-/-^*  HC (4), CFC (4) | Genotype x Extinction  Interaction *p* = 0.0027;  Genotype *p* = 0.0416;  CFC *p* = 0.2027  Bonferroni posttest:  HC *Trpm2^+/+^* vs. *Trpm2^-/-^* *p* = 0.7252;  CFC *Trpm2^+/+^* vs. *Trpm2^-/-^* *p* = 0.0059;  HC *Trpm2^+/+^* vs. CFC *Trpm2^+/+^* *p* = 0.0133 | Genotype x Extinction  Interaction F_(1,13)_ = 13.72;  Genotype F_(1,13)_ = 5.110;  CFC F_(1,13)_ = 1.799 |
| 3b  *c-Fos* | Two-way ANOVA | *Trpm2^+/+^*  HC (5), CFC (4);  *Trpm2^-/-^*  HC (4), CFC (4) | Genotype x Extinction  Interaction *p* = 0.0013;  Genotype *p* = 0.0020;  CFC *p* = 0.0456  Bonferroni posttest:  HC *Trpm2^+/+^* vs. *Trpm2^-/-^* *p* = 0.9984;  CFC *Trpm2^+/+^* vs. *Trpm2^-/-^* *p* = 0.0005;  HC *Trpm2^+/+^* vs. CFC *Trpm2^+/+^* *p* = 0.0026 | Genotype x Extinction  Interaction F_(1,13)_ = 16.65;  Genotype F_(1,13)_ = 14.88;  CFC F_(1,13)_ = 4.887 |
| 3b  *Arc* | Two-way ANOVA | *Trpm2^+/+^*  HC (3), CFC (3);  *Trpm2^-/-^*  HC (3), CFC (3) | Genotype x Extinction  Interaction *p* = 0.4232;  Genotype *p* = 0.4712;  CFC *p* = 0.1791  Bonferroni posttest:  HC *Trpm2^+/+^* vs. *Trpm2^-/-^* *p* > 0.9999;  CFC *Trpm2^+/+^* vs. *Trpm2^-/-^* *p* = 0.6820;  HC *Trpm2^+/+^* vs. CFC *Trpm2^+/+^* *p* = 0.9689 | Genotype x Extinction  Interaction F_(1,8)_ = 0.7122;  Genotype F_(1,8)_ = 0.5718;  CFC F_(1,8)_ = 2.168 |
| 3b  *Egr1* | Two-way ANOVA | *Trpm2^+/+^*  HC (3), CFC (3);  *Trpm2^-/-^*  HC (3), CFC (3) | Genotype x Extinction  Interaction *p* = 0.6377;  Genotype *p* = 0.5526;  CFC *p* = 0.5200  Bonferroni posttest:  HC Trpm2^+/+^ vs. Trpm2^-/-^ *p* = 0.9997;  CFC Trpm2^+/+^ vs. Trpm2^-/-^ *p* = 0.8596;  HC Trpm2^+/+^ vs. CFC Trpm2^+/+^ *p* = 0.9992 | Genotype x Extinction  Interaction F_(1,8)_ = 0.2395;  Genotype F_(1,8)_ = 0.3842;  CFC F_(1,8)_ = 0.4528 |
| 3c  *Npas4* | Two-way ANOVA | *Trpm2^+/+^*  HC (5), FE (4);  *Trpm2^-/-^*  HC (5), FE (5) | Genotype x Extinction  Interaction *p* = 0.0007;  Genotype *p* = 0.0876;  FE *p* = 0.0323  Bonferroni posttest:  HC *Trpm2^+/+^* vs. *Trpm2^-/-^* *p* = 0.6089;  FE *Trpm2^+/+^* vs. *Trpm2^-/-^* *p* = 0.0051;  HC *Trpm2^+/+^* vs. FE *Trpm2^+/+^* *p* = 0.0024 | Genotype x Extinction  Interaction F_(1,15)_ = 17.84;  Genotype F_(1,15)_ = 3.340;  FE F_(1,15)_ = 5.563 |
| 3c  *c-Fos* | Two-way ANOVA | *Trpm2^+/+^*  HC (6), FE (4);  *Trpm2^-/-^*  HC (5), FE (5) | Genotype x Extinction  Interaction *p* = 0.0278;  Genotype *p* = 0.0366;  FE *p* = 0.0223  Bonferroni posttest:  HC *Trpm2^+/+^* vs. *Trpm2^-/-^* *p* > 0.9999;  FE *Trpm2^+/+^* vs. *Trpm2^-/-^* *p* = 0.0359;  HC *Trpm2^+/+^* vs. FE *Trpm2^+/+^* *p* = 0.0192 | Genotype x Extinction  Interaction F_(1,16)_ = 5.856;  Genotype F_(1,16)_ = 5.200;  FE F_(1,16)_ = 6.397 |
| 3c  *Arc* | Two-way ANOVA | *Trpm2^+/+^*  HC (6), FE (4);  *Trpm2^-/-^*  HC (5), FE (3) | Genotype x Extinction  Interaction *p* = 0.0130;  Genotype *p* = 0.2918;  FE *p* = 0.0446  Bonferroni posttest:  HC *Trpm2^+/+^* vs. *Trpm2^-/-^* *p* > 0.9999;  FE *Trpm2^+/+^* vs. *Trpm2^-/-^* *p* = 0.0244;  HC *Trpm2^+/+^* vs. FE *Trpm2^+/+^* *p* = 0.0910 | Genotype x Extinction  Interaction F_(1,16)_ = 7.806;  Genotype F_(1,16)_ = 4.748;  FE F_(1,16)_ = 1.189 |
| 3c  *Egr1* | Two-way ANOVA | *Trpm2^+/+^*  HC (6), FE (4);  Trpm2^-/-^  HC (5), FE (5) | Genotype x Extinction  Interaction *p* = 0.0068;  Genotype *p* = 0.0217;  FE *p* = 0.0606  Bonferroni posttest:  HC *Trpm2^+/+^* vs. *Trpm2^-/-^* *p* > 0.9999;  FE *Trpm2^+/+^* vs. *Trpm2^-/-^* *p* = 0.0093;  HC *Trpm2^+/+^* vs. FE *Trpm2^+/+^* *p* = 0.0148 | Genotype x Extinction  Interaction F_(1,16)_ = 9.636;  Genotype F_(1,16)_ = 6.472;  FE F_(1,16)_ = 4.074 |
| 4c  CFC | Two-way RM ANOVA | Vehicle (14);  FFA (13) | Drug x Shock  Interaction *p* = 0.5298;  Drug *p* = 0.0635;  Shock *p* < 0.0001  Bonferroni posttest:  Baseline Vehicle vs. FFA *p* > 0.9999;  Shock1 Vehicle vs. FFA *p* = 0.0993;  Shock2 Vehicle vs. FFA *p* = 0.3654;  Shock3 Vehicle vs. FFA *p* > 0.9999 | Drug x Shock  Interaction F_(3,75)_ = 0.7430;  Drug F_(1,25)_ = 3.772;  Shock F_(3,75)_ = 79.56 |
| 4d  2 h after CFC | Unpaired two-tailed t test | Vehicle (14);  FFA (13) | Vehicle vs. FFA *p* = 0.3850 | t_(25)_ = 0.8843 |
| 4d  24 h after CFC | Unpaired two-tailed t test | Vehicle (14);  FFA (13) | Vehicle vs. FFA *p* = 0.1246 | t_(25)_ = 1.589 |
| 4e  Extinction | Two-way RM ANOVA | Vehicle (14);  FFA (13) | Drug x Extinction  Interaction *p* = 0.9162;  Drug *p* = 0.1362;  Extinction *p* = 0.0050  Bonferroni posttest:  E1 Vehicle vs. FFA *p* = 0.9221;  E2 Vehicle vs. FFA *p* > 0.9999;  E3 Vehicle vs. FFA *p* > 0.9999;  E4 Vehicle vs. FFA *p* > 0.9999;  E5 Vehicle vs. FFA *p* > 0.9999;  E6 Vehicle vs. FFA *p* > 0.9999;  E7 Vehicle vs. FFA *p* = 0.7072 | Drug x Extinction  Interaction F_(6,150)_ = 0.3375;  Drug F_(1,25)_ = 2.371;  Extinction F_(6,150)_ = 3.248 |
| 5c  Extinction | Unpaired two-tailed t test | Vehicle (11);  FFA (12) | E1 Vehicle vs. FFA *p* = 0.3630;  E2 Vehicle vs. FFA *p* = 0.0696;  E3 Vehicle vs. FFA *p* = 0.0013;  E4 Vehicle vs. FFA *p* = 0.0035;  E5 Vehicle vs. FFA *p* = 0.0258;  E6 Vehicle vs. FFA *p* = 0.0052;  E7 Vehicle vs. FFA *p* = 0.0318 | t_(21)_ = 0.9298;  t_(21)_ = 1.912;  t_(21)_ = 3.695;  t_(21)_ = 3.289;  t_(21)_ = 2.399;  t_(21)_ = 3.119;  t_(21)_ = 2.300 |
| 5c  Overall | Two-way RM ANOVA | Vehicle (11);  FFA (12) | Drug x Extinction  Interaction *p* = 0.0400;  Drug *p* = 0.0058;  Extinction *p* < 0.0001 | Drug x Extinction  Interaction F_(6,126)_ = 2.281;  Drug F_(1,21)_ = 9.451;  Extinction F_(6,126)_ = 38.44 |
| 5d  24 h after extinction | Unpaired two-tailed t test | Vehicle (3);  FFA (4) | Vehicle vs. FFA *p* = 0.2247 | t_(5)_ = 1.385 |
| 5e  *Npas4* | Unpaired two-tailed t test | Vehicle (3);  FFA (4) | Vehicle vs. FFA *p* = 0.0084 | t_(5)_ = 4.206 |
| 5f  *c-Fos* | Unpaired two-tailed t test | Vehicle (3);  FFA (4) | Vehicle vs. FFA *p* = 0.0315 | t_(5)_ = 2.959 |
| 5g  *Arc* | Unpaired two-tailed t test | Vehicle (3);  FFA (4) | Vehicle vs. FFA *p* = 0.0746 | t_(5)_ = 2.246 |
| 5h  *Egr1* | Unpaired two-tailed t test | Vehicle (3);  FFA (4) | Vehicle vs. FFA *p* = 0.0011 | t_(5)_ = 6.762 |
